# Do Post-Translational Modifications Influence Protein Aggregation in Neurodegenerative Diseases: A Systematic Review

**DOI:** 10.3390/brainsci10040232

**Published:** 2020-04-11

**Authors:** Larissa-Nele Schaffert, Wayne G. Carter

**Affiliations:** School of Medicine, University of Nottingham, Royal Derby Hospital Centre, Uttoxeter Road, Derby DE22 3DT, UK; larissaschaffert@yahoo.de

**Keywords:** neurodegenerative diseases, neurotoxicity, post-translational modifications, protein aggregates

## Abstract

The accumulation of abnormal protein aggregates represents a universal hallmark of neurodegenerative diseases (NDDs). Post-translational modifications (PTMs) regulate protein structure and function. Dysregulated PTMs may influence the propensity for protein aggregation in NDD-proteinopathies. To investigate this, we systematically reviewed the literature to evaluate effects of PTMs on aggregation propensity for major proteins linked to the pathogenesis and/or progression of NDDs. A search of PubMed, MEDLINE, EMBASE, and Web of Science Core Collection was conducted to retrieve studies that investigated an association between PTMs and protein aggregation in seven NDDs: Alzheimer’s disease (AD), Parkinson’s disease (PD), Huntington’s disease (HD), amyotrophic lateral sclerosis (ALS), spinocerebellar ataxias, transmissible spongiform encephalopathy, and multiple sclerosis. Together, 1222 studies were identified, of which 69 met eligibility criteria. We identified that the following PTMs, in isolation or combination, potentially act as modulators of proteinopathy in NDDs: isoaspartate formation in Aβ, phosphorylation of Aβ or tau in AD; acetylation, 4-hydroxy-2-neonal modification, *O*-GlcNAcylation or phosphorylation of α-synuclein in PD; acetylation or phosphorylation of TAR DNA-binding protein-43 in ALS, and SUMOylation of superoxide dismutase-1 in ALS; and phosphorylation of huntingtin in HD. The potential pharmacological manipulation of these aggregation-modulating PTMs represents an as-yet untapped source of therapy to treat NDDs.

## 1. Introduction

Neurodegenerative diseases (NDDs) are a major cause of global morbidity and mortality in the elderly, and, with an ever-rising prevalence, represent one of the greatest health challenges of the 21st century. NDDs encompass heterogeneous cerebral proteinopathies, characterised by a progressive loss of vulnerable neurons such that patients present with broad clinical sequelae that includes motor, behavioural, and cognitive deficits [1,2,3]. At autopsy, NDDs can be characterised histopathologically via hallmark intra- or extracellular accumulations of degradation-resistant protein aggregates concentrated to certain brain regions (Table 1). These protein aggregates interfere with neuronal function, and presumably induce toxicity that ultimately drives cell death [2].

Although the pathogenesis of Huntington’s disease (HD) and spinocerebellar ataxias (SCAs), as well as rare familial forms of other NDDs are influenced by gene mutations that affect protein structure and function, the majority of NDDs arise from a multifactorial idiopathic aetiology. 

Numerous drug therapies to treat NDDs have entered clinical trials over the last two decades, but their low success rates underscore substantial gaps in understanding of the molecular mechanisms that drive neurodegeneration in sporadic diseases [1,2,3]. Consequently, there are no specific curative treatments available to reverse or even halt the progression of brain pathology in NDDs, and all therapeutics licensed for treatment merely work at a symptomatic level. Furthermore, the number of patients with these age-related diseases is rising due to increased longevity, and this carries an enormous social and economic global burden. It is therefore crucial to elucidate the molecular mechanisms that trigger protein aggregation and subsequent neurotoxicity in order to identify potential targets for drug development, to resist or possibly reverse disease progression. 

### 1.1. Alzheimer’s Disease

Alzheimer’s disease (AD) is the most common NDD and accounts for 60–80% of all cases of dementia [27,30]. More than 30 million people are currently suffering from AD, and this number is estimated to increase to 115 million by 2050 [31]. Clinically, patients predominantly present with a decline in cognitive function that initially manifests as episodic short-term memory loss, but as the disease progresses, visuospatial, behavioural, and psychiatric disturbances follow and eventually lead to the inability to perform activities of daily living [32]. Diagnosing AD relies heavily on patient history and cognitive function tests such as the Mini Mental State Examination or the Montreal Cognitive Assessment, but screening for plasma and CSF biomarkers and brain imaging have proven helpful ancillary methods [1,33]. Nevertheless, macroscopic confirmation of AD is provided from post-mortem examination, and typified by widespread cortical atrophy primarily within the frontotemporal lobes (including the hippocampus) that leads to an enlargement of the lateral ventricles [1,2,34]. Microscopically, AD pathology is characterised by extracellular plaque deposits of aggregated amyloid-beta peptide (Aβ) as well as intracellular neurofibrillary tangles (NFTs) primarily composed of hyperphosphorylated fibrils of microtubule-associated protein tau [2,34].

Aβ peptide is primarily composed of 42–43 amino acids formed from proteolytic cleavage of the transmembranous amyloid precursor protein (APP) [34,35]. Two proteases that act on APP, a β-secretase and γ-secretase, yield the Aβ_40_ and Aβ_42_ isoforms, respectively. Alternatively, APP may be processed by α-secretase via the non-amyloidogenic pathway [35,36,37]. While Aβ is believed to play a number of physiological roles, including regulation of synaptic activity [36,37], in AD, it acquires a toxic gain-of-function, undergoes oligomerisation and aggregation, eventually forming insoluble, fibrillar, senile plaques that are central to neurotoxicity and neurodegeneration [2,38,39]. 

Human tau protein exists in six isoforms of between 352 and 441 amino acids encoded by the *MAPT* gene on chromosome 17q21.31. It is localised predominantly to neuronal axons, where its primary function is the stabilisation of microtubules and regulation of neuronal transport [40,41,42]. In AD, abnormally phosphorylated tau adopts an altered conformation that hinders its binding to microtubules and promotes its self-assembly (aggregation) into paired helical filaments (PHFs), the primary component of NFTs [40,41,42]. It is these tau aggregates and Aβ aggregates that have been the subject of targeted therapies [42].

### 1.2. Parkinson’s Disease

Approximately 1% of individuals over the age of 60 years and close to 5% of the population aged 80 or older suffer from Parkinson’s disease (PD), the second most common NDD and most common movement disorder [43]. PD is characterised by a progressive loss of dopaminergic neurons in the subcortical basal ganglia; specifically, within the substantia nigra pars compacta (SNpc) [1,2,43,44,45]. Since this midbrain region plays a crucial role in fine-tuning motor circuits and facilitating movement, PD manifests clinically with a pathognomonic triad of bradykinesia, rigidity, and resting tremor [1,45]. In addition to nigral degeneration, the primary pathological feature of PD is the presence of intraneuronal proteinaceous inclusions termed Lewy bodies (LBs), primarily composed of α-synuclein [2,46,47].

α-synuclein is a cytosolic 140 amino acid protein encoded by the *SNCA* gene on chromosome 4q21. Although widely expressed within neurons, it is abundant at presynaptic terminals, indicative of a role in synaptic signalling. A number of rare *SNCA* point mutations trigger dominant familial (early-onset) forms of PD. Fibrillar forms of α-synuclein have been identified within LBs that accumulate in hereditary and sporadic forms of PD [46,47,48,49]. Oligomeric and/or fibrillar aggregates of α-synuclein may be cytotoxic and trigger neuronal degeneration.

### 1.3. Amyotrophic Lateral Sclerosis

Amyotrophic lateral sclerosis (ALS) is a fatal, incurable NDD characterised by the loss of upper and lower motor neurons. ALS typically presents with symptoms of bulbar and spinal dysfunction including muscle weakness, wasting and spasticity, and ultimately, patients may become paralysed and die from respiratory failure [2,50,51]. Pathologically, both sporadic and familial ALS are characterised by marked cytoplasmic protein aggregates in degenerating neurons that harbour ubiquitinated forms of Trans-activation response DNA-binding protein 43 (TDP-43) and the antioxidant enzyme, superoxide dismutase 1 (SOD1) [10,50,51]. 

TDP-43 is a 414 amino acid protein encoded by the *TARDBP* gene on chromosome 1 and is normally nuclear resident and involved in the regulation of RNA metabolism. In ALS, cytoplasmic, ubiquitinated, hyperphosphorylated, and truncated forms of TDP-43 accumulate as protein aggregates [52,53,54]. These cytoplasmic aggregates are neurotoxic, produce ALS-like phenotypes, and contribute to a loss of nuclear (functional) TDP-43 [52,53,54,55].

Superoxide dismutase 1 (SOD1) is a 153-amino acid copper- and zinc-dependent metalloenzyme encoded by the *SOD1* gene on chromosome 21q22.11. SOD1 functions to scavenge highly reactive superoxide radicals by catalysing their conversion to hydrogen peroxide and molecular oxygen [56]. SOD1 is predominantly cytoplasmic, although nuclear, lysosomal, and mitochondrial residences have also been reported [57]. In SOD1-mediated familial ALS, mutations in the *SOD1* gene sequence are thought to affect post-translational processing, rendering the protein prone to misfolding, aggregation, and formation of neuronal inclusion bodies. Misfolded SOD1 is also found in cytoplasmic inclusions in patients with sporadic ALS, as well as other proteins including TDP-43 [58]; suggesting a common pathophysiological mechanism for hereditary and idiopathic ALS. 

### 1.4. Huntington’s Disease

Huntington’s disease (HD) is a rare, autosomal dominant NDD with an average age of onset of 40 years [59]. HD is caused by an expansion of the CAG repeat (≥36) in the Huntingtin (*Htt*) gene on chromosome 4p16.3, that when translated produces an elongated polyglutamine (polyQ) stretch in the Htt protein [59]. The formation of aggregated Htt within neuronal intranuclear inclusion bodies is a histopathological hallmark of HD [60]. Additionally, the degeneration of GABAergic medium spiny neurons in the striatum that project to other regions of the basal ganglia and thereby modulate central motor circuitries, is central to HD pathology [61]. This is reflected in the clinical picture of HD such that patients present with severe motoric abnormalities including prominent ‘dance-like’ involuntary movements termed chorea [62]. With disease progression, other brain regions undergo degeneration, with an associated array of additional psychiatric, behavioural, and cognitive symptoms.

Htt is a large, 3144-amino acid, monomeric protein required for embryonic neurogenesis that controls a number of nuclear and cytoplasmic homeostatic functions including regulation of synaptic activity [63,64]. In HD, the expanded polyQ stretch of the protein induces production of N-terminal Htt fragments that are prone to misfold and form amyloid-like structures [65,66,67,68]. Furthermore, polyQ tracts have a tendency to form β-pleated sheets and this conformational change directly increases the aggregation propensity of Htt [66,67,68]. These changes result in the assembly of oligomeric structures and eventually the formation of Htt-positive intracellular inclusion bodies that disrupt cellular homeostasis and cause neuronal degeneration [69].

### 1.5. Spinocerebellar Ataxias

Spinocerebellar ataxias (SCAs) are a heterogeneous group of >27 different autosomal dominant NDDs that share the phenotypical core feature of ataxia and are characterized by degenerative and atrophic changes in the central nervous system, primarily affecting the cerebellum [70]. The most common SCAs belong to the polyQ repeat diseases and are caused by CAG expansion mutations in a number of different genes [71]. 

### 1.6. Transmissible Spongiform Encephalopathy

Transmissible spongiform encephalopathies (TSEs), also known as prion diseases, are progressive NDDs that typically present with variable symptomology including dementia and ataxia [72]. They can be classified according to their genetic, acquired, and sporadic aetiology, with the most common TSE being sporadic Creutzfeldt-Jakob disease [23]. TSEs typically present with a pathology that involves neuronal vacuolation (spongiform change), widespread neuronal death, and subsequent gliotic scarring in cerebral grey matter secondary to the accumulation of aberrant, self-propagating prion protein scrapie (PrP^sc^) [73]. Diagnostic criteria as well as ancillary tests such as electroencephalography or genetic testing facilitate diagnosis, but only at autopsy can a definitive diagnosis be confirmed [74]. Due to the propagating nature of TSEs and lack of disease-modifying or curative treatments, after clinical onset, the disease course is usually short with a median survival of 5 months [75].

The prion gene *PRNP* on chromosome 20 encodes a human prion glycoprotein, PrP^c^, of 253 amino acids localised to pre- and postsynaptic terminals in the CNS [76,77,78]. Its precise physiological function has been debated and is considered non-essential [79]: indicative that a toxic gain-of-function rather than loss-of-function of PrP^c^ underlies neurotoxicity in prion diseases [80]. In TSEs, PrP^c^ misfolds and is partially converted from an α-helical to β-sheet structure that is resistant to proteolytic clearance, PrP^sc^ [78,81]. Misfolded PrP^sc^ accumulates in synaptic and axonal processes and forms fibrillar aggregates that are capable of inducing neuronal apoptosis in vitro and in vivo [80,82]. Pathology is thought to be propagated when abnormal PrP^sc^ converts normal PrP^c^ into PrP^sc^ via an autocatalytic mechanism [83].

### 1.7. Multiple Sclerosis

Multiple Sclerosis (MS) is traditionally classed as a neuroinflammatory disease caused by autoimmune-mediated axonal demyelination within the CNS [84,85]. Clinical presentation is variable since focal inflammatory lesions may affect any CNS structure, and the diagnostic process is therefore based on a combination of clinical history and examination, brain imaging, and blood screening [84]. There are four MS subtypes: relapsing remitting (RRMS), progressive relapsing (PRMS), primary progressive (PPMS) and secondary progressive (SPMS), and each has a unique therapeutic approach and prognosis [84,85].

While most subjects (≈85%) are diagnosed with RRMS at symptomatic onset, the majority of patients will progress to development of highly debilitating SPMS [85]. Post-mortem analyses of MS patients have demonstrated degenerative lesions and significant cerebral atrophy indicative of a NDD. Furthermore, the identification of proteinaceous, oligomeric aggregates, that include Aβ, tau, and APP, as well as deposits in neuronal somata composed of aggregated bassoon (Bsn) protein, may represent a link between neuroinflammation and neurodegeneration in MS [8,12,13].

Bsn is a large, 3926 amino acid, scaffold protein that is part of the presynaptic cytoskeletal matrix, and has multiple roles in mediating synaptic function [12,86]. In MS, Bsn is mis-localised and accumulates in neuronal cells, to induce neurotoxicity and neurodegeneration [12].

### 1.8. Protein Aggregation in Neurodegeneration

Protein misfolding and accumulation of toxic aggregates has emerged as a central theme of paradigmatic NDDs [87]. Protein turnover is an orchestrated process controlled via a balance of protein synthesis and degradation. Cells are equipped with efficient protein quality control mechanisms such as the ubiquitin-proteasome system and chaperone-mediated autophagy that are able to eliminate aberrant or misfolded proteins [87,88]. However, the capacity or indeed fidelity of these degradative pathways may be compromised with a corresponding accumulation of damaged, misfolded, or aggregated proteins, and associated pathology. Hence the formation of protein aggregates may serve as an initiating step in cellular dysfunction in NDDs, and it is therefore critical to understand the molecular mechanisms that alter the protein aggregation of these NDD-associated proteins. 

### 1.9. Post-Translational Modifications and NDDs

The chemical modifications of proteins during or after their biosynthesis via the covalent attachment of functional groups or proteolytic cleavage at specific amino acids are collectively termed protein post-translational modifications (PTMs). PTMs can be mediated by enzymatic or non-enzymatic means and may be reversible or irreversible. Over 600 different PTMs have been demonstrated experimentally [89]. This plethora of PTMs diversifies the proteome by modulating the structural and functional properties of proteins. Due to their pivotal role in regulating cellular processes and roles in ageing, protein PTMs may need to be strictly controlled, such that dysregulation of PTMs could contribute to disease pathogenesis or progression [90,91].

The major risk factor for developing NDD is age, and this may also correlate with disturbed PTM homeostasis. One could hypothesise that as an individual ages the likelihood of dysregulation of PTMs increases, and if this leads to aberrant modification of susceptible proteins then protein misfolding and aggregation, neurotoxicity, and ultimately neurodegeneration may ensue. Against this backdrop, the identification of the PTMs that drive dysregulated protein function proffer a therapeutic window if the formation of toxic protein aggregates could be obviated. Indeed, recent animal studies have demonstrated that therapies that influence protein PTMs provide a viable means to modulate proteinopathy and associated neurodegeneration [91,92].

The aim of this systematic research was to provide a comprehensive, unbiased analysis of the PTMs that have been reported to affect the aggregation propensity of proteins implicated in the pathology of AD, PD, ALS, HD, SCAs, TSEs, and MS. Mapping of the patterns and number of studies demonstrating aggregation-modulating PTMs will improve our understanding of the extent and site-specificity of aggregation-inducing PTMs, and thereby provide an insight into novel therapeutic targets that may limit pathogenesis and/or disease progression.

## 2. Methods

A systematic review of the literature was carried out according to the Preferred Reporting Items for Systematic Reviews and Meta-Analyses (PRISMA).

### 2.1. Search Strategy

From the 7th to 17th September 2019, a systematic electronic database search was conducted on MEDLINE (OvidSP), EMBASE (OvidSP), Web of Science Core Collection, and PubMed to retrieve all experimental studies investigating the effect of PTMs on protein aggregation in the pre-specified NDDs. A combination of controlled vocabulary (MeSH) and free-text keywords were used for each of the four concepts and search terms included: (a) neurodegenerative diseases, nerve degeneration, neurodegenerat *, Alzheimer’s disease, Parkinson’s disease, amyotrophic lateral sclerosis, Huntington’s disease, spinocerebellar ataxia, transmissible spongiform encephalopathy, and multiple sclerosis; (b) protein aggregation, aggreg *; (c) beta-amyloid, tau, alpha-synuclein, TDP-43, superoxide dismutase 1, huntingtin, prions, protein aggregate, inclusion bodies; (d) post-translational modifications, protein processing. The full search strategy for MEDLINE is provided as Supplementary Data S1. Additional studies were identified through bibliography screening of relevant review articles and hand-searching of relevant articles.

### 2.2. Eligibility Criteria

All search results (n = 1222) were exported into Endnote (Clarivate Analytics) and Excel (Microsoft) for duplicate removal (automatic deduplication and manual checking) and title/abstract screening to identify studies deemed appropriate to the pre-specified inclusion criteria. Included articles were original studies directly investigating the effect of PTMs at residues of human proteins central to pathology in AD, PD, ALS, HD, SCAs, TSEs, or MS for which the PTM had an effect on aggregation behaviour. Studies were excluded if they focused on proteins or NDDs other than those pre-defined, or were published in a language other than English, or performed with non-human proteins or tissue, or were review articles, editorials, or conference abstracts.

### 2.3. Data Acquisition and Analysis

Eligible publications were read in full and information for the following variables collated as a data extraction spreadsheet: NDD; protein investigated; type of PTM; site of PTM; peptide or protein source; in vitro or in vivo study; assay method for aggregation; overall study findings and conclusion; authors and year of publication.

## 3. Results

**Figure 1:**
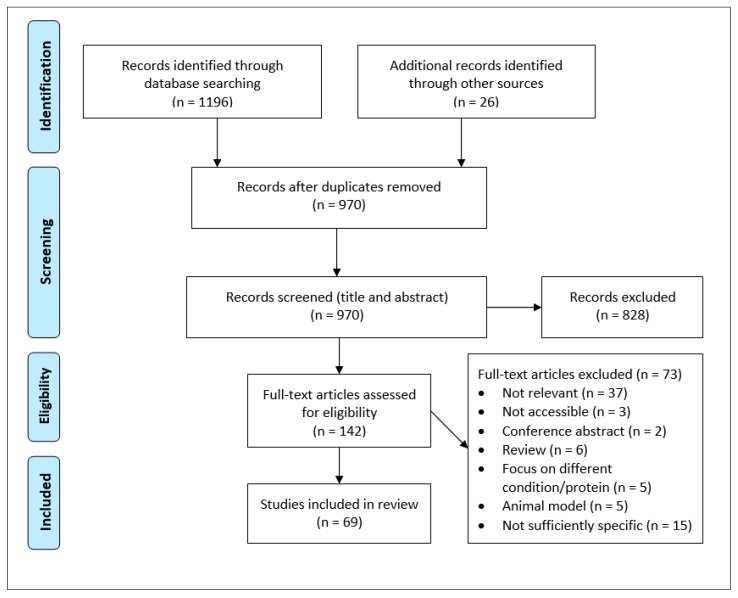
Preferred reporting items for systematic reviews and meta-analyses (PRISMA) flow chart detailing the stages of study retrieval and selection [94].

A total of 1196 articles were identified from the database search and an additional 26 potentially relevant papers yielded from hand-searching key papers. Duplicate articles were removed, and then 166 papers excluded based upon title screening, yielding 804 papers for abstract review. Further screening led to the exclusion of 662 articles and left a final 142 papers for full-text assessment. Of these, 73 studies did not meet the pre-defined eligibility criteria and were rejected based on the following grounds: off-topic (n = 37), full-text not accessible (n = 3), conference abstract (n = 2), review article (n = 6), focus on condition/protein not pre-specified in the inclusion criteria (n = 5), animal model (n = 5), lack of specificity (n = 15). The remaining 69 articles fulfilled the inclusion criteria and were included in the final analysis (Figure 1).

Of the 69 included studies, the majority were focused upon PTMs and protein aggregation in AD (n = 28) and then PD (n = 20), 10 studies considered ALS, 7 HD, and 2 studies each examined SCAs and TSEs, respectively. No studies investigating the effect of PTMs on protein aggregation in the context of MS were identified from this search. The most commonly employed method to assess aggregation behaviour was Thioflavin T (ThT) fluorescence for quantitation of in vitro formation of amyloid-like fibrils [93]. Other studies that examined aggregation included the use of Western blotting, immunohistochemistry, sedimentation assays, atomic force microscopy, and transmission electron microscopy. The results for each NDD-related protein are presented and stratified according to effects on: (1) aggregation in general; (2) formation of oligomeric species; (3) formation of fibrillar aggregates; and (4) formation of amorphous aggregates. 

### 3.1. Alzheimer’s Disease

#### 3.1.1. Aβ PTMs and Propensity for Aggregation

Eleven studies focused upon aggregation behaviour of Aβ. Two studies investigated isoaspartate modification (collectively covering residues D1, D7, and D23) and both reported that it had a pro-aggregation effect, with enhanced formation of Aβ oligomers as well as fibrillar aggregates [95,96]. A similar pro-aggregation effect was observed after N-terminal pyroglutamylation at E3 of Aβ [97]. The effect of phosphorylation on aggregation propensity of Aβ varied for different residues: specifically, S8 phosphorylation increased the formation of oligomeric and high molecular weight species [98,99], while phosphorylation at S26 had an overall inhibitory effect on the formation of large aggregates through stabilising intermediate oligomers [100]. Reduced transition from oligomer to fibril was also observed for *N*-homocysteinylation at K16 and K28 [101]. The results relating to nitration at Y10 were inconclusive: two studies reported a decrease of Aβ aggregation propensity [102,103], whereas one study reported a pro-aggregation effect [104]. Lastly, glycation at R5 and K16 reduced the formation of fibrillar aggregates [105]. A detailed overview of the effect of each PTM on the aggregation behaviour of Aβ is presented in Table 2.

**Figure 2:**
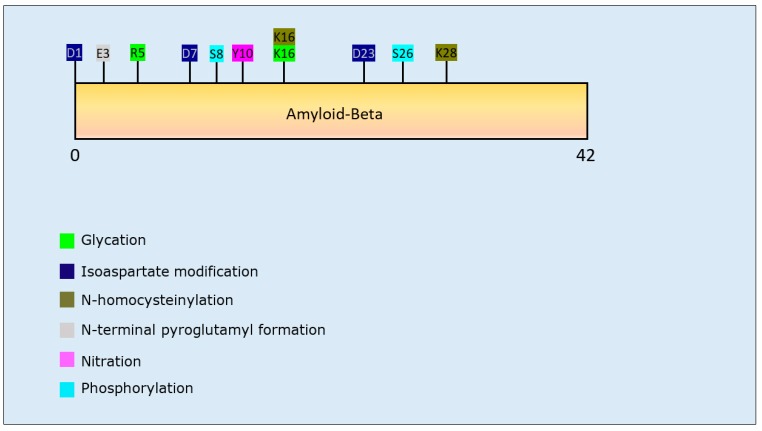
Schematic representation of beta-amyloid-42 peptide illustrating PTMs and respective amino acid residues.

Interestingly, all PTMs that affected Aβ aggregation were within the first 28 residues of the peptide (Figure 2), leaving the 29–40 C-terminal section less prone to structural alterations.

#### 3.1.2. Tau PTMs and Propensity for Aggregation

The majority of studies that have investigated PTMs of tau and protein aggregation have focused upon acetylation. Collectively, the results of acetylation analyses have been equivocal; with a demonstration of increased aggregation after acetylation at K280/K281 [106,107,108], but with acetylation at multiple lysine residues, including K280/281, also reported to decrease aggregation and the fibrillation rate of tau [109,110]. Furthermore, K321 acetylation alone or in combination with acetylation at other lysine residues decreased the level of tau aggregates [110,111].

**Figure 3:**
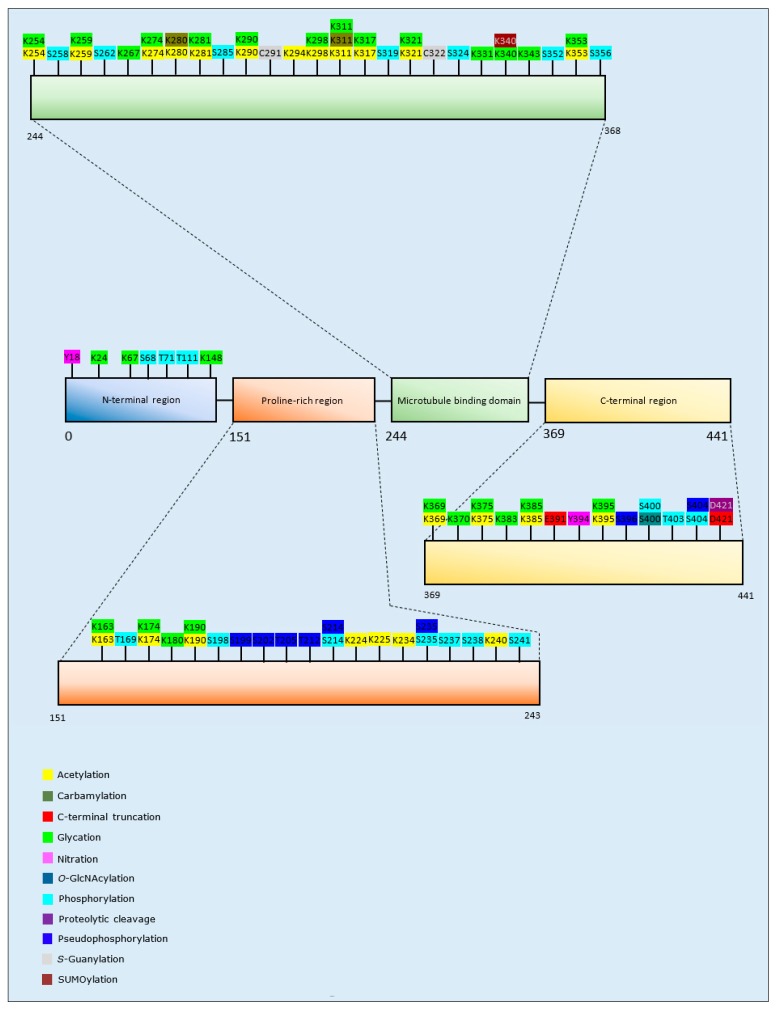
Schematic representation of tau protein illustrating PTMs and respective amino acid residues.

Carbamylation, C-terminal truncation, glycation, proteolytic cleavage, pseudo-phosphorylation (replacement of a phospho-acceptor amino acid with a negatively charged amino acid to mimic phosphorylation), and SUMOylation all primarily increased tau assembly into aggregates [112,113,114,115,116,117,118], with the exception of pseudo-phosphorylation at S235 that significantly reduced tau aggregation [116]. *O*-GlcNAcylation, *S*-guanylation, and methylation decreased aggregation [119,120,121]. Likewise, site-specific tau nitration at Y18 and Y394 resulted in the formation of fewer and/or shorter tau filaments compared to the unmodified protein [122]. The effect of phosphorylation of tau and aggregation varied for different isoforms: phosphorylation of 4R2N and 3R1N at specific residues increased tau aggregation, while phosphorylation of 4R0N and 3R2N tau isoforms decreased their aggregation [114]. Additionally, one study identified an ‘acetylation-phosphorylation switch’, whereby acetylation of K321 was able to prevent phosphorylation at S324, a common PTM observed in tau inclusions in patients with AD [111]. A summary of the PTMs of tau that influence aggregation propensity are included as Table 3, and Figure 3.

### 3.2. Parkinson’s Disease

#### α-Synuclein PTMs and Propensity for Aggregation

Twenty studies investigated the effects of PTMs on the PD-related protein, α-synuclein. Collectively, acetylation of the N-terminal region of α-synuclein reduced aggregation in four out of five studies [123,124,125,126], and with one study that reported that this PTM increased the propensity of α-synuclein to aggregate [127]. Adenylylation at T33, T54, and T75 reduced α-synuclein aggregation [128]. Glycation at multiple lysine residues increased α-synuclein aggregation and formation of stable oligomers [129]. Increased oligomerization but reduced aggregation was triggered after SUMOylation at K96 and K102 [130]. 4-hydroxy-2-neonal (HNE) modification at H50 resulted in enhanced formation of oligomers, which in two studies reduced the formation of fibrillar aggregates [131,132,133]. *O*-GlcNAcylation decreased formation of aggregates, and favoured the formation of oligomers after modification at T81 and S87, or when *O*-GlcNAcylation was performed across the whole protein [134,135]. By contrast, α-synuclein *O*-GlcNAcylated at T72 formed markedly fewer oligomers compared to the non-modified protein [136]. *O*-GlcNAcylation at T72 also inhibited S129 phosphorylation [136]; a PTM that increased the formation of α-synuclein aggregates [137,138]. Lastly, nitration of α-synuclein displayed conflicting results regarding effects upon α-synuclein aggregation [131,139,140,141,142]. Although all of these studies demonstrated that nitration increased oligomerisation, this was associated with a reduction or inability to form fibrils [131,139,141]. A summary of the PTMs that influence aggregation propensity of α-synuclein are shown in Table 4.

**Figure 4:**
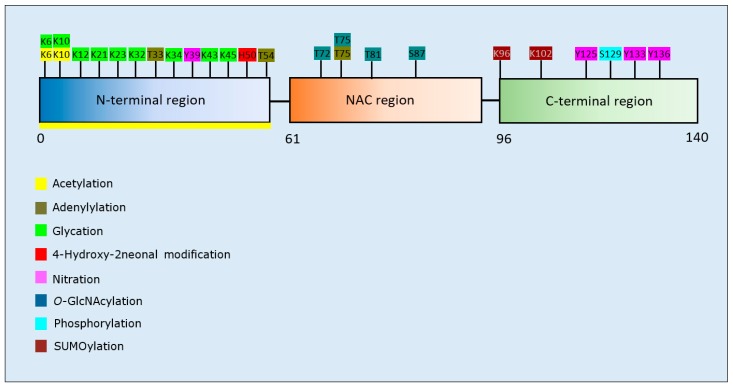
Schematic representation of α-synuclein illustrating PTMs and respective amino acid residues.

Aggregation-related modifications of α-synuclein cluster to discrete regions of the protein, dependent upon the type of PTM (Figure 4). More specifically, acetylation, glycation, and HNE modifications were confined to the N-terminal region, *O*-GlcNAcylation targeted the NAC region, and SUMOylation, phosphorylation, and nitration were predominantly at the C-terminal region.

### 3.3. Amyotrophic Lateral Sclerosis

#### 3.3.1. TAR DNA-Binding Protein 43 PTMs and Propensity for Aggregation

**Figure 5:**
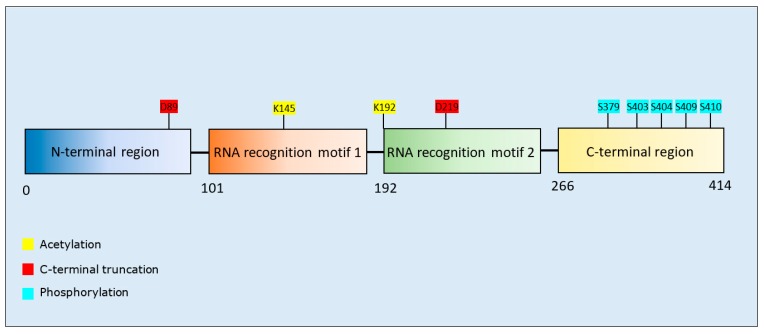
Schematic representation of TDP-43 illustrating PTMs and respective amino acid residues.

Acetylation of K145 and K192 increased aggregation of TDP-43 [143,144]. C-terminal fragmentation at D89 and D219 produced aggregation-prone TDP-43 fragments (residues 90–414 or residues 220–414, respectively) [145]. Phosphorylation of TDP-43 was detected at multiple sites, including S409/S410, and increased TDP-43 aggregation in two studies [146,147], but decreased the aggregation of C-terminal fragments of TDP-43 [148]. Phosphorylation at multiple serine residues (including S409 and S410) also induced a protective (reduction of aggregation) rather than pathogenic (pro-aggregation) effect [149]. C-terminal fragmentation at D219 produced a protein more prone to phosphorylation at S409/S410 [145], and similarly, acetylation at K145 promoted the accumulation of TDP-43 aggregates hyperphosphorylated at S409/S410 [144] (Table 5). TDP-43 phosphorylation arose primarily at residues clustered within the glycine-rich C-terminus (Figure 5); a protein region susceptible to formation of amyloid-like fibrils [150,151].

#### 3.3.2. SOD1 PTMs and Propensity for Aggregation

**Figure 6:**
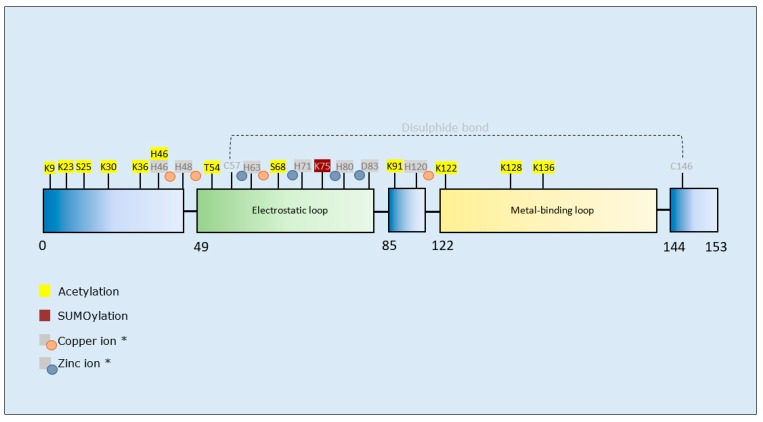
Schematic representation of SOD1 illustrating PTMs and respective amino acid residues. * Due to their importance for SOD1 structural stability, metal-binding sites, and residues involved in disulphide bond formation are also marked.

The agent employed for acetylation of SOD1 in vitro as well as the site(s) of acetylation influenced the type of aggregates formed [152]. SUMOylation at K75 increased aggregation of mutant (and wild-type) SOD1 [153,154] (Table 6 and Figure 6).

### 3.4. Huntington’s Disease

#### Htt PTMs and Propensity for Aggregation

**Figure 7:**
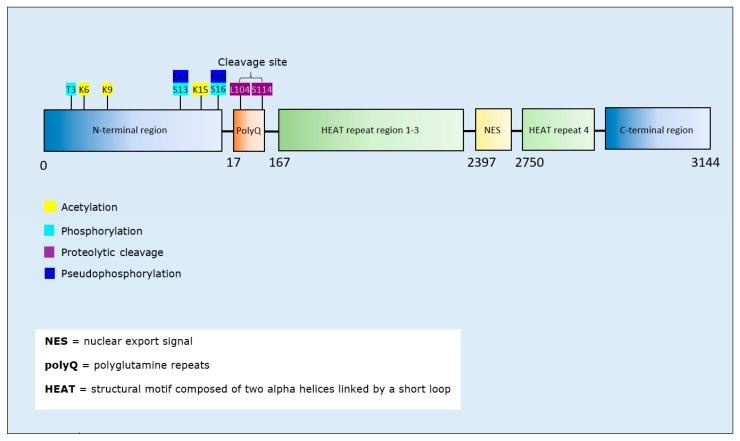
Schematic representation of huntingtin illustrating PTMs and respective amino acid residues.

Acetylation of Htt decreased the formation aggregated fibrils [155]. Htt phosphorylation at T3 decreased Htt aggregation [156,157,158], as did phosphorylation at S13 and/or S16 [159,160]. Proteolytic cleavage of mutant Htt generated fragments with an increased tendency to aggregate into nuclear and cytoplasmic inclusion bodies [161] (Table 7). Residues targeted by acetylation, phosphorylation, or proteolytic cleavage were confined to the N-terminal region and polyQ stretch of Htt (Figure 7); regions that play a critical role in Htt aggregation behaviour [161,162].

### 3.5. Spinocerebellar Ataxias

#### Ataxins PTMs and Propensity for Aggregation

**Figure 8:**
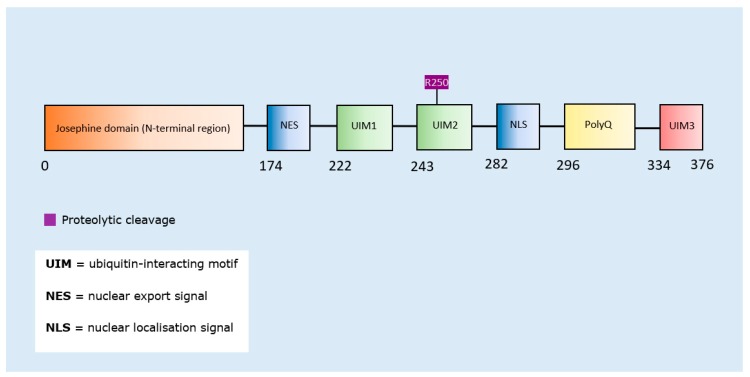
Schematic representation of ataxin-3 illustrating PTMs and respective amino acid residues.

SUMOylation of ataxin-1 (across the whole protein) enhanced aggregation [163], as did proteolytic cleavage of ataxin-3 [164] (Table 8). Since SUMOylation of ataxin-1 was not confined to a specific amino acid residue, a schematic representation was only produced for ataxin-3. The proteolytic cleavage site was mapped to the second ubiquitin-interacting motif domain of the protein (Figure 8).

### 3.6. Transmissible Spongiform Encephalopathies

#### Prion Protein PTMs and Propensity for Aggregation

**Figure 9:**
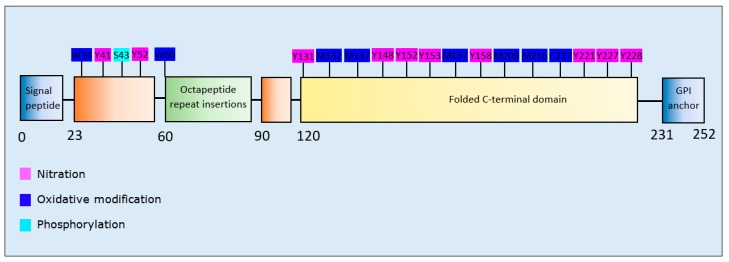
Schematic representation of PrP^c^ illustrating PTMs and respective amino acid residues.

Oxidation and nitration at multiple residues of PrP^c^ increased aggregation propensity with enhanced formation of fibrillar as well as amorphous aggregates [165]. Phosphorylation at S43 of PrP^c^ enhanced its aggregation, with a tendency of the phosphorylated form to produce large fibrillar and fewer amorphous aggregates than unmodified PrP^c^ [166] (Table 9). PrP^c^ residues susceptible to nitrative and oxidative modifications were primarily clustered within the folded C-terminal domain of the protein (Figure 9). 

### 3.7. Multiple Sclerosis

**Bsn:** Bsn protein was identified only recently as a protein aggregate that may contribute to neurotoxicity in MS [12], and an examination of the influence of PTMs upon Bsn aggregation has yet to be undertaken.

**Aβ and tau:** The detection of oligomeric aggregates of Aβ and tau, and APP within brain tissue from MS patients [8,13] suggests that for some NDDs, a common subset of potentially toxic protein accumulations exist. The PTMs that influence protein aggregation of Aβ and tau, are detailed in Table 2 and Table 3, respectively, and Figure 2 and Figure 3, respectively. Whether similar PTMs are relevant to the Aβ and tau deposited in brain tissue of MS patients has yet to be determined.

## 4. Discussion

This review considered PTMs that may influence pathogenic protein aggregation in NDDs. Evidence indicating a role for PTMs in a NDD tended to reflect the prevalence of the disease: with most studies focused upon AD or PD, with relatively sparse literature coverage of PTMs in SCAs or TSEs, and no studies identified that investigated the PTM of Bsn protein in MS. Furthermore, for many of the studies of the PTMs, only a single report of the effect of the PTM has been published. However, for some of the PTMs, at least two independent studies have considered aggregation behaviour in response to a PTM at the same amino acid, and these have been discussed further:

**Aβ isoaspartate:** Results from Table 2 indicate that isoaspartate formation has a pro-aggregation effect on Aβ. Consistent with a role for this PTM in AD, the levels of isoaspartate in Aβ was significantly higher in brain tissue from patients with AD compared with age-matched controls [167]. Isoaspartate is a non-enzymatic PTM that forms in peptides or proteins from asparagine deamination or aspartic acid isomerisation [168,169]. Isoaspartate formation influences protein structure and function since it results in a kink in the peptide backbone and the addition of a methylene group. Protein levels of isoaspartate are normally restricted in vivo via the action of the enzyme, protein L-isoaspartyl methyltransferase (PIMT) [168,169]. However, for peptides or proteins that become extracellular, including Aβ, inaccessibility to cytosolic or nuclear PIMT activity renders them susceptible to ongoing isoaspartate formation. A therapeutic intervention that prevents the formation of isoaspartate within Aβ and thereby limits its pro-aggregation effect may be of benefit in AD, perhaps through targeted repair of Aβ or increased activity of PIMT. 

**Aβ phosphorylation**: The results of Table 2 suggest that there are divergent effects of phosphorylation on Aβ aggregation; such that phosphorylation of S8 stimulated Aβ aggregation, whereas phosphorylation at S26 decreased aggregation. Hence, total phosphorylation of Aβ is unlikely to be an informative marker of aggregation propensity and toxicity. Moreover, site-specific phosphorylation may need to be considered for therapeutic interventions: agents that, for example, enhance phosphatase activity or inhibit kinase activity at S8, or conversely inhibit phosphatase activity and promote kinase activity at S26 may be useful for limiting pathogenic aggregation.

**Tau phosphorylation:** Although hyperphosphorylation of tau as a trigger for the formation of NFTs and progression of AD has been reported [170], evidence collated in this review suggests that tau phosphorylation and aggregation is only specific to certain tau isoforms (Table 3). Indeed, phosphorylation of the tau isoforms 4R0N and 3R2N resulted in decreased tau aggregation [114]. By contrast, phosphorylation or pseudo-phosphorylation of the longest tau isoform, 4R2N, increased tau aggregation [114,116,117]. Clinical treatments for AD that have utilised kinase inhibitors to prevent tau hyperphosphorylation and aggregation have had limited success [171], and future studies will need to consider those tau isoforms that exhibit increased propensity to aggregate after phosphorylation, as well as the influence of site-specific phosphorylations.

**α-synuclein acetylation**: Four of five studies reported reduced aggregation of α-synuclein after acetylation within the N-terminal region (Table 4). Acetylation of α-synuclein interferes with internal hydrogen-bonding and induces an α-helical rather than β-pleated sheet structure, and this may be the mechanism for decreased aggregation [124,125]. In contrast, a single study reported increased α-synuclein aggregation after acetylation [127]. Hence, collectively, there may be a beneficial therapeutic anti-aggregation effect upon α-synuclein through targeted N-terminal acetylation.

**α-synuclein 4-hydroxy-2-neonal (HNE) modification:** NDDs are associated with cellular redox stress that can lead to increased lipid peroxidation and formation of the α,β-unsaturated hydroxyalkenal, HNE, capable of covalently adducting α-synuclein [133]. Adduction of α-synuclein by HNE reduced the tendency of α-synuclein to undergo fibrillar aggregation (Table 4) [131,133]. However, whether this could be exploited as a protective modification is equivocal, since HNE modification decreased aggregation by stabilising oligomeric intermediates, and α-synuclein oligomers may also be toxic to neurons [172,173].

**α-synuclein *O*-GlcNAcylation:** Protein glycosylation as *O*-GlcNAcylation arises from enzymatic *O*-linked addition of β-N-acetyl-glucosamine at serine or threonine residues and is involved in a number of homeostatic mechanisms. All three studies undertaken to date reported decreased α-synuclein aggregation after *O*-GlcNAcylation [134,135,136]. Furthermore, interplay between PTMs was reported, with an inhibitory effect of α-synuclein T72 *O*-GlcNAcylation upon α-synuclein S129 phosphorylation [136]; a PTM that normally enhances α-synuclein aggregation and promotes neuropathology [137,138]. Collectively, these studies suggest a benefit of *O*-GlcNAcylation that could be manipulated by therapeutic intervention. However, total levels of *O*-GlcNAcylation were increased in post-mortem tissue from three PD patients compared to control subjects, although specific levels of *O*-GlcNAcylation of α-synuclein were not determined [174]. 

**α-synuclein phosphorylation:** Phosphorylation at S129 of α-synuclein has a pro-aggregation effect, is detected in LBs, and is associated with proteinopathy in PD [137,138,174]. Hence, there may be therapeutic value in manipulating the levels of this phosphorylation. To that end, a transgenic mouse model of α-synucleinopathy with enhanced dephosphorylation at S129 displayed reduced aggregation and symptomatic improvement [91]. 

**TDP-43 acetylation:** Acetylated TDP-43 has been recovered within spinal cord of patients with ALS [144], indicative of its pathological relevance. In support of this supposition, two studies demonstrated enhanced aggregation of TDP-43 following its acetylation (Table 5). Since the acetylated residues (K145 and K192) are within the nucleic acid binding regions (Figure 5), this PTM may reduce RNA-binding of TDP-43 and promote the accumulation of dysfunctional TDP-43 aggregates [144]. Thus, preventing TDP-43 acetylation may provide a new approach for treatment of ALS.

**Superoxide Dismutase 1 SUMOylation:** SUMOylation of mutant or wild-type SOD1 by SUMO1 and SUMO3 at K75 enhanced its aggregation [153,154]. Thus, preventing SUMOylation of SOD1 may be beneficial to limit progression of fALS. Certainly, inhibition of mutant SOD1 SUMOylation at K75 has proven a successful approach to prevent SOD1 aggregation in neuronal cells [175], and therefore underpins a rationale for translation to animal models.

**Huntingtin phosphorylation:** Preclinical models of HD and HD patient brain samples display lower phosphorylation levels at T3 of huntingtin compared with controls [158]. Htt phosphorylation at T3 as well as at S13/16 inhibits the formation of aggregates, potentially serving as a protective mechanism against proteinopathic changes (Table 7). Consistent with this proposal, restored N-terminal Htt phosphorylation reversed neurotoxicity in a model of HD [176]. Therefore, modification of the levels of Htt phosphorylation represent a promising target for therapeutic development. Additionally, Htt phosphorylation at T3 prevents phosphorylation at S13/S16 [177]. Nevertheless, since both phosphorylation at T3 and S13/S16 decrease Htt aggregation, it will be of interest to delineate their potential cumulative effects upon Htt aggregation. 

A summary of the PTMs whose manipulations may be of therapeutic benefit are included in Table 10.

### 4.1. Study Limitations

The majority of studies investigating the roles of PTMs for proteins that contribute to NDDs have been undertaken in vitro, and it is therefore difficult to make direct inferences to in vivo effects. Furthermore, while we have compiled and summarised the primary literature that has examined the effects of PTMs upon protein aggregation, these studies invariably only consider individual PTMs, and therefore will not reflect the multiple PTMs experienced within a dynamic in vivo system. Hence, analysis of site-specific PTMs by, for example, mass spectrometric means, only provides a snapshot of site occupancy at that juncture. Indeed, PTMs co-exist in vivo and may act in a permissive, reciprocal, antagonising, potentiating, or even synergistic manner to influence overall protein structure and function. Additionally, to consider functional relevance of each PTM to support therapeutic targeting, it is also important to consider the stoichiometry associated with each PTM, and this has not yet been addressed in most of the in vitro or in vivo studies. 

Our study has also only focussed upon the influence of the PTM upon protein aggregation, and for which measurements of aggregation were conducted by different means. The majority of studies have utilised ThT fluorescent assays, but other protein separation and imaging systems were also used–refer to Supplementary Data, S2. Furthermore, differences between biological models, protein sources (for example, synthetic or recombinant peptides or proteins), and assay techniques, could account for some of the conflicting studies that describe the effects of PTMs on aggregation behaviour. Furthermore, we have tried to consider different aggregation elements: oligomers, fibrillary, and amorphous aggregates, but some studies have demonstrated reduced aggregate formation via increased stability of oligomeric species. Hence, one must interpret results with caution due to the unresolved debate as to whether oligomers and/or mature aggregates are the primary toxic species [178]. While both oligomers and larger aggregates are known to adopt β-sheet conformations, Aβ and α-synuclein oligomers can be arranged in antiparallel β-pleated sheets, whereas fibrillar aggregates display a parallel β-sheet structure [179,180]. Hence, differences in secondary structures, certainly for Aβ, may ultimately influence neurotoxicity [181]. Lastly, the formation of toxic oligomers or aggregates may not only be a consequence of increased protein aggregation propensity, but also arise from dysfunctional protein degradation pathways. 

### 4.2. Summary and Conclusions

A more comprehensive understanding of the molecular mechanisms that trigger pathogenesis or progression of NDDs is a prerequisite to developing new treatment options. The results of this review highlight that multiple PTMs can alter aggregation potential and thereby contribute to proteinopathies. The future development of therapies to modify PTM profiles for key NDD-related proteins may provide an as yet untapped source of novel drug treatments for NDDs. 

## Figures and Tables

**Table 1:** Overview of neuropathology, approximate global prevalence rates, and frequency of genetic and sporadic forms of major neurodegenerative diseases [3,4,5,6,7,8,9,10,11,12,13,14,15,16,17,18,19,20,21,22,23,24,25,26,27,28,29].

NDD	Commonly Mutated Proteins	Primary Region of Damage	Compartment of Aggregate Deposition	Aggregate-Forming Proteins	Global Prevalence	Sporadic Cases	Familial Cases
AD	APP, presenilins	Cortex, hippocampus	Extracellular, intracytoplasmic	Aβ (plaques), tau (tangles)	593:100,000	>98%	<2%
PD	α-synuclein, LRRK2	Substantia nigra, cortex	Intracytoplasmic	α-synuclein (Lewy bodies)	1–2:1000	>90%	<10%
HD	Htt	Striatum, basal ganglia	Intranuclear, intracytoplasmic	Htt	1:10,000	3%	97%
ALS	TDP-43, SOD1, c9orf72	Spinal motor neurons, motor cortex	Intracytoplasmic	SOD1, TDP-43	5:100,000	90–95%	5–10%
MS	-	Basal ganglia, brainstem	Intracytoplasmic, extracellular	Aβ, tau, APP, bassoon protein	30.1:100,000	80–90%	10–20%
SCAs	ATX1, ATX2, ATX3, CACNA1A, ATX7, TBP, ATN1	Cerebellum, brainstem	Intranuclear	Atrophin-1, ataxins	3:100,000	No data	No data
TSEs	PrP	Cortex, brainstem, thalamus, cerebellum	Extracellular	PrP	1–2:1,000,000	85–90%	10–15%

Abbreviations: APP, Amyloid precursor protein; LRRK2, Leucine-rich repeat kinase-2; Htt, Huntingtin; TDP-43, TAR DNA-binding protein 43; c9orf72, Chromosome 9 open reading frame 72; SOD1, Superoxide dismutase 1; ATX, ataxins; CACNA1A, Voltage-gated calcium channel subunit α1A; TBP, TATA-binding protein; ATN1, atrophin 1; PrP, Prion protein.

**Table 2:** Summary of β-amyloid PTMs and propensity for protein aggregation.

Post-Translational Modification	Residues Modified	Author & Year	Aggregation	Formation of Oligomers	Formation of Fibrillar Aggregates	Formation of Amorphous Aggregates
Glycation	R5, K16	Emendato et al., 2018 [105]	Decrease	-	Decrease	-
Isoaspartate formation	D23	Shimizu et al., 2002 [96]	Increase	-	Increase	-
	D1, D7, D23	Fossati et al., 2013 [95]	Increase	Increase	Increase	-
*N*-Homocysteinylation	K16, K28	Khodadadi et al., 2012 [101]	Decrease	Increase	Decrease	-
N-terminal pyroglutamylation	N-terminal E3	Schilling et al., 2006 [97]	Increase	Increase	Increase	-
Nitration	Y10	Kummer et al., 2011 [104]	Increase	Increase	Increase	-
	Y10	Zhao et al., 2015 [102]	Decrease	Decrease	Decrease	-
	Y10	Guivernau et al., 2016 [103]	Decrease	Increase	Decrease	-
Phosphorylation	S8	Jamasbi et al., 2017 [98]	Increase	-	Increase	-
	S8	Kumar et al., 2011 [99]	Increase	Increase	Increase	-
	S26	Kumar et al., 2016 [100]	Decrease	Increase	Decrease	-

**Table 3:** Summary of Tau PTMs and propensity for protein aggregation.

Post-Translational Modification	Isoform and Residues Modified	Author & Year	Aggregation	Formation of Oligomers	Formation of Fibrillar Aggregates	Formation of Amorphous Aggregates
Acetylation	4R2N: K280/K281	Trzeciakiewicz et al., 2017 [106]	Increase	-	Increase	-
	4R2N: K163/K174/K190/K224/K234/K240/K254/K280/K281/K290/K311/K375/K385/K395	Ferreon et al., 2018 [109]	Decrease	-	Decrease	-
	4R0N: K321, K259/K290/K321/K353, K290/K321, K274	Carlomagno et al., 2017 [111]	Decrease	-	Decrease	-
	4R2N: K163, K174, K224, K225, K234, K240, K259, K274, K280, K290/K321, K294, K298, K317, K353, K369	Kamah et al., 2014 [110]	Decrease	-	Decrease	-
	4R2N: K280	Haj-Yahya and Lashuel, 2018 [107]	Increase	Increase	Decrease	-
	4R, Tau-K18: K163/K280/K281/K369	Cohen et al., 2011 [108]	Increase	-	Increase	-
Carbamylation	4R2N: K311, K280, K311/K280	KrishnaKumar et al., 2018 [112]	Increase	-	Increase	-
C-terminal Truncation	4R2N: D421, E391	Yin and Kuret, 2006 [113]	Increase	-	Increase	-
Glycation	4R2N: K67, K148, K163, K180, K190, K259, K267, K274, K281, K290, K298, K311, K317, K321, K331, K340, K343, K353, K369, K370, K375, K383, K385, K395	Liu et al., 2016 [114]	Increase	-	Increase	-
	3R2N: K24, K163, K174, K180, K190, K254, K259, K267, K311, K343, K353, K369, K385	Liu et al., 2016 [114]	Increase	-	Increase	-
Methylation	4R2N: Multiple residues*	Funk et al., 2014 [121]	Decrease	-	Decrease	-
Nitration	4R2N: Y18, Y394	Reynolds et al., 2005 [122]	Decrease	-	Decrease	-
*O*-GlcNAcylation	4R2N: S400	Yuzwa et al., 2014 [119]	Decrease	-	Decrease	-
Phosphorylation	4R2N: S68, T169, S214, S262, S285, S319, S356, T403	Liu et al., 2016 [114]	Increase	-	Increase	-
	3R1N: T71	Liu et al., 2016 [114]	Increase	-	Increase	-
	4R0N: T111, S198, S214, S237, S238, S241, S258, S324, S352, S356, S400, S404	Liu et al., 2016 [114]	Decrease	-	Decrease	-
	3R2N: S235, S237, S324	Liu et al., 2016 [114]	Decrease	-	Decrease	-
Proteolytic cleavage	4R2N: D421	Mead et al., 2016 [115]	Increase	-	-	Increase
Pseudo-phosphorylation	4R2N: S199, S199/S202/T205, T212, S214, T212/S214, S396/S404,	Necula and Kuret, 2004 [116]	Increase	-	Increase	-
	4R2N: S235	Necula and Kuret, 2004 [116]	Decrease	-	Decrease	-
	4R2N: T212	Chang et al., 2011 [117]	Increase	-	Increase	-
*S*-Guanylation	3R2N: C291	Yoshitake et al., 2016 [120]	Decrease	Decrease	Decrease	-
	4R2N: C291, C322	Yoshitake et al., 2016 [120]	Decrease	Decrease	Decrease	-
SUMOylation	4R2N: K340	Luo et al., 2014 [118]	Increase	-	-	-

* Tau methylation at multiple residues across the protein.

**Table 4:** Summary of α-synuclein PTMs and propensity for protein aggregation.

Post-Translational Modification	Residues Modified	Author & Year	Aggregation	Formation of Oligomers	Formation of Fibrillar Aggregates	Formation of Amorphous Aggregates
Acetylation	N-terminus	Bartels et al., 2014 [123]	Decrease	-	Decrease	-
	N-terminus	Kang et al., 2012 [124]	Decrease	-	Decrease	-
	N- terminus	Bu et al., 2017 [125]	Decrease	Decrease	Decrease	-
	N-terminus	Birol et al., 2019 [127]	Increase	-	Increase	-
	N-terminus, K6, K10	Oliveira et al., 2017 [126]	Decrease	Decrease	Decrease	-
Adenylylation	T33, T54, T75	Sanyal et al., 2019 [128]	Decrease	-	Decrease	-
Glycation	K6, K10, K12, K21, K23, K32, K34, K43, K45	Vicente et al., 2017 [129]	Increase	Increase	Decrease	Increase
4-Hydroxy-2-neonalModification	H50, and other Lys residues	Qin et al., 2006 [133]	Decrease	Increase	Decrease	-
	H50, and other Lys residues	Xiang et al., 2013 [131]	Decrease	Increase	Decrease	-
	H50	Xiang et al., 2015 [132]	-	Increase	-	-
Nitration	Y39, Y125, Y133/Y136	Burai et al., 2015 [139]	Decrease	Increase	Decrease	Increase
	Y39, Y125, Y133, Y136	Liu et al., 2011 [140]	-	Increase	-	Increase
	Y39, Y125, Y133, Y136	Hodara et al., 2004 [141]	Increase	-	-	-
	Y39, Y125, Y133, Y136	Souza et al., 2000 [142]	Increase	Increase	-	-
	Y39/Y125/Y133/136	Xiang et al., 2013 [131]	Decrease	Increase	Decrease	-
*O*-GlcNAcylation	T72	Levine et al., 2019 [134]	Decrease	-	Decrease	-
	T75	Levine et al., 2019 [134]	Decrease	-	Decrease	-
	T81	Levine et al., 2019 [134]	Decrease	Increase	Decrease	-
	S87	Levine et al., 2019 [134]	Decrease	Increase	Decrease	-
	T72/T75/T81	Levine et al., 2019 [134]	Decrease	-	Decrease	-
	Muliple sites *	Zhang et al., 2017 [135]	Decrease	Increase	Decrease	-
	T72	Marotta et al., 2015 [136]	Decrease	Decrease	Decrease	-
Phosphorylation	S129	Fujiwara et al., 2002 [137]	Increase	Increase	Increase	-
	S129	Samuel et al., 2016 [138]	Increase	-	Increase	-
SUMOylation	K96, K102	Krumova et al., 2011 [130]	Decrease	Increase	Decrease	-

* α-synuclein *O*-GlcNAcylation at multiple residues across the entire protein was investigated.

**Table 5:** Summary of TDP-43 PTMs and propensity for protein aggregation.

Post-Translational Modification	Residues Modified	Author & Year	Aggregation	Formation of Oligomers	Formation of Fibrillar Aggregates	Formation of Amorphous Aggregates
Acetylation	K145	Wang et al., 2017 [143]	Increase	-	-	-
	K145, K192	Cohen et al., 2015 [144]	Increase	-	-	-
C-terminal fragmentation	D89, D219	Zhang et al., 2009 [145]	Increase	-	-	-
Phosphorylation	S379, S403, S404, S409, S410, S403/S404, S409/S410, S379/S403/S404, S379/S409/S410, S403/S404/S409/S410	Li et al., 2011 [149]	Decrease	-	-	-
	S409/S410	Carlomagno et al., 2014 [146]	Increase	-	Increase	-
	S379, S403/404, S409, S410, S409/S410	Hasegawa et al., 2008 [147]	Increase	Increase	Increase	-
	S409/S410	Brady et al., 2011 [148]	Decrease	-	-	-

**Table 6:** Summary of SOD1 PTMs and propensity for protein aggregation.

Post-Translational Modification	Residues Modified	Author & Year	Aggregation	Formation of Oligomers	Formation of Fibrillar Aggregates	Formation of Amorphous Aggregates
Acetylation	K23/K30/K36/H46/K91/K122/K128/K136 (BP *)	Rasouli et al., 2017 [152]	-	-	Decrease	-
	K36/K128 (BT *)	Rasouli et al., 2017 [152]	-	-	Decrease	Increase
	K23/S25/K30/K36/K91/K122/K128/K136 (PM *)	Rasouli et al., 2017 [152]	-	-	Decrease	Increase
	K9 (CA*)	Rasouli et al., 2017 [152]	-	-	Increase	-
	K9/K23/K30/K36/K91/K122/K136/T54/S68 (GA *)	Rasouli et al., 2017 [152]	-	-	Decrease	Increase
	K23/S25/K30/K36/K122/K128/K136 (SA *)	Rasouli et al., 2017 [152]	-	-	Increase	-
SUMOylation	K75 (SUMO3) ^†^	Niikura et al., 2014 [153]	Increase	-	-	-
	K75 (SUMO1) ^‡^	Fei et al., 2006 [154]	Increase	-	-	-

* Abbreviations: BP, 3,3’,4,4’-biphenyltetracarboxylic dianhydride; BT, benzophenone-3,3’,4,4’-tetracarboxylic dianhydride; PM, pyromellitic dianhydride; CA, citraconic anhydride; GA, glutaric anhydride; SA, succinic anhydride. ^†^ Mutant SOD-1 was used in this study. ^‡^ Wild-type and mutant SOD-1 were used in this study.

**Table 7:** Summary of huntingtin PTMs and propensity for protein aggregation.

Post-Translational Modification	Residues Modified	Author & Year	Aggregation	Formation of Oligomers	Formation of Fibrillar Aggregates	Formation of Amorphous Aggregates
Acetylation	K6, K9, K15 ^‡^	Chaibva et al., 2016 [155]	Decrease		Decrease	-
Phosphorylation	T3 *	Chiki et al., 2017 [156]	Decrease	-	Decrease	-
	T3	Ansaloni et al., 2014 [157]	Decrease	Decrease	Decrease	-
	T3 *	Cariulo et al., 2017 [158]	Decrease	-	-	-
	S13, S16, S13/S16 ^†^	DeGuire et al., 2018 [159]	Decrease	Increase	Decrease	-
Pseudo-phosphorylation	S13, S16 ^†^	DeGuire et al., 2018 [159]	Decrease	Increase	Decrease	-
	S13/S16 *	Gu et al., 2009 [160]	Decrease	-	Decrease	-
Proteolytic Cleavage	Cleavage site between residue 104 and 114 *	Lunkes et al., 2002 [161]	Increase	-	-	-

* Mutant huntingtin protein was used in this study. ^†^ Wild-type and mutant huntingtin protein were used in this study. ^‡^ Wild-type and truncated huntingtin protein were used in this study.

**Table 8:** Summary of ataxins PTMs and propensity for protein aggregation.

Post-Translational Modification	Residues Modified	Author & Year	Aggregation	Formation of Oligomers	Formation of Fibrillar Aggregates	Formation of Amorphous Aggregates
SUMOylation (ataxin-1)	Multiple residues	Ryu et al., 2010 [163]	Increase	-	-	-
Proteolytic Cleavage (ataxin-3)	N-terminal region cleavage near residue 250	Haacke et al., 2006 [164]	Increase	-	-	-

**Table 9:** Summary of PrP^c^ PTMs and propensity for protein aggregation.

Post-Translational Modification	Residues Modified	Author & Year	Aggregation	Formation of Oligomers	Formation of Fibrillar Aggregates	Formation of Amorphous Aggregates
Oxidative modification and Nitration	Oxidation: W34, W60, M157, M209, M216, C217, M132/M137	Dear et al., 2007 [165]	Increase	-	Increase	Increase
	Nitration: Y41, Y41/Y52, Y131, Y148, Y152, Y153, Y158, Y221, Y227/Y228		Increase	-	Increase	Increase
Phosphorylation	S43	Giannopoulos et al., 2009 [166]	Increase	-	Increase	Decrease

**Table 10:** Summary of PTMs with therapeutic potential and suggested pharmacological interventions.

Protein	PTM	Residues	Schematic Representation of Modification	Suggested Pharmacological Intervention
Aβ	Isoaspartate modification	D1, D7, D23	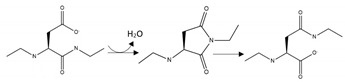	Inhibition
Aβ	Phosphorylation	S8	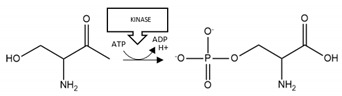	Inhibition
Aβ	Phosphorylation	S26	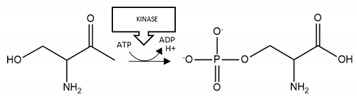	Enhancement
αS	Acetylation	K6, K10, N-terminal region	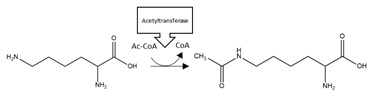	Enhancement
αS	HNE modification	H50	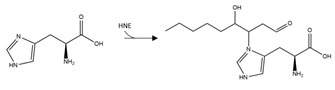	Inhibition
αS	*O*-GlcNacylation	T72, T75, T81, S87	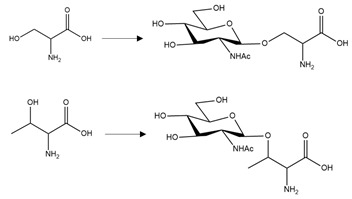	Enhancement
αS	Phosphorylation	S129	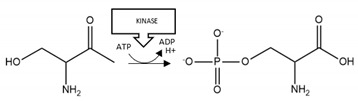	Inhibition
TDP-43	Acetylation	K145, K192	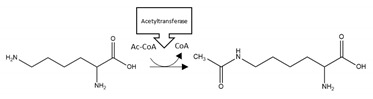	Inhibition
SOD1	SUMOylation	K75	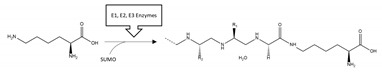	Inhibition
HTT	Phosphorylation	T3, S13, S16	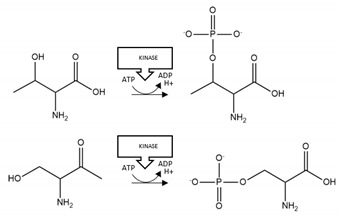	Enhancement

Abbreviations: αS, α-synuclein.

## Supplementary Materials

The following are available online at https://www.mdpi.com/2076-3425/10/4/232/s1.
