# Supplementary material for: Do Post-Translational Modifications Influence Protein Aggregation in Neurodegenerative Diseases: A Systematic Review
Source: Brain Sci. 2020 Apr 11;10(4):232. doi: 10.3390/brainsci10040232 (PMC7226274; doi:10.3390/brainsci10040232)
Supplement: Supplementary file 1 [file brainsci-10-00232-s001.zip › Supplementary Data S1.docx]

Supplementary Data S1: MEDLINE Search Strategy

|  | 1. Neurodegenerative Diseases/ or Nerve Degeneration/ |
| --- | --- |
|  | Neurodegenerat*.mp |
|  | Parkinson's disease.mp. or Parkinson Disease/ |
|  | Alzheimer's disease.mp. or Alzheimer Disease/ |
|  | Huntington's disease.mp. or Huntington Disease/ |
|  | Amyotrophic Lateral Sclerosis.mp. or Amyotrophic Lateral Sclerosis/ |
|  | Spinocerebellar ataxia.mp. or Spinocerebellar Ataxias/ |
|  | Multiple Sclerosis.mp. or Multiple Sclerosis/ |
|  | Prion Diseases/ or Encephalopathy, Bovine Spongiform/ or transmissible spongiform encephalopathy.mp |
|  | **1** or **2** or **3** or **4** or **5** or **6** or **7** or **8** or **9** |
|  | Protein Aggregation, Pathological/ or protein aggregation.mp |
|  | Aggreg*.mp |
|  | **11** or **12** |
|  | Alpha-synuclein.mp. or alpha-Synuclein/ |
|  | Amyloid beta-Peptides/ or Plaque, Amyloid/ or beta-amyloid.mp. or Amyloid beta-Protein Precursor/ |
|  | Huntingtin Protein/ or huntingtin.mp. |
|  | Superoxide Dismutase.mp. or Superoxide Dismutase/ |
|  | Tau Protein.mp. or tau Proteins/ |
|  | DNA-Binding Proteins/ or tdp-43.mp |
|  | Prions/ or Prion Proteins/ |
|  | Protein aggregates.mp. or Protein Aggregates/ or Inclusion Bodies/ |
|  | **14** or **15** or **16** or **17** or **18** or **19** or **20** or **21** |
|  | Post-translational modification.mp. or Protein Processing, Post-Translational/ |
|  | **10** and **13** and **22** and **23** |
|  | Limit **24** to English language |
|  | Limit **25** to humans |
