# Supplementary material for: Do Post-Translational Modifications Influence Protein Aggregation in Neurodegenerative Diseases: A Systematic Review
Source: Brain Sci. 2020 Apr 11;10(4):232. doi: 10.3390/brainsci10040232 (PMC7226274; doi:10.3390/brainsci10040232)
Supplement: Supplementary file 1 [file brainsci-10-00232-s001.zip › Supplementary Data S2.docx]

Supplementary Data S2. Study Methods and Protein Source

| Author & Year | Study Methods for Assessment of Aggregation | Protein Source |
| --- | --- | --- |
| Beta-Amyloid | | |
| Emendato *et al.*, 2018 [105] | ThT, AFM, CD | Synthetic (in-house and commercial peptide) |
| Shimizu *et al.*, 2002 [96] | ThT, CD, EM | Synthetic |
| Fossati *et al.,* 2013 [95] | ThT, WB, CD, TEM, dot-blot | Synthetic |
| Khodadadi *et al.*, 2012 [101] | ThT, CD, EM | Commercial Peptide |
| Schilling *et al.*, 2006 [97] | ThT, FC | Synthetic |
| Kummer *et al.*, 2011 [104] | ThT, WB, dot-blot | Synthetic |
| Zhao *et al.,* 2015 [102] | ThT, TEM | Synthetic |
| Guivernau *et al.*, 2016 [103] | ThT, WB, EM, AFM, CD | Synthetic |
| Jamasbi *et al.*, 2017 [98] | ThT, CD | Synthetic |
| Kumar *et al.*, 2011 [99] | ThT, Congo Red, CD, TEM, WB | Synthetic |
| Kumar *et al.*, 2016 [100] | ThT, Congo Red, TEM, AFM | Synthetic |
| Tau | | |
| Trzeciakiewics *et al.*, 2017 [106] | ThT, heparin-induced sedimentation assay, EM, CD, SDS-PAGE | Recombinant |
| Ferreon *et al.*, 2018 [109] | ThT, heparin-induced sedimentation assay | Recombinant |
| Carlomagno *et al.*, 2017 [111] | ThS, IHC, WB, EM | Recombinant |
| Kamah *et al.*, 2014 [110] | ThT, heparin-induced sedimentation assay, EM | Recombinant |
| Haj-Yahya and Lashuel, 2018 [107] | ThS, TEM, heparin-induced sedimentation assay | Recombinant, semisynthetic |
| Cohen et al., 2011 [108] | ThT, sedimentation assay, EM | Recombinant |
| KrishnaKumar *et al.,* 2018 [112] | ThT, Congo Red, TEM, AFM, turbidity measurements | Recombinant |
| Yin and Kuret, 2005 [113] | Thiazine Red aggregation, TEM | Recombinant |
| Liu et al., 2016 [114] | ThT, heparin-induced sedimentation assay | Recombinant |
| Funk *et al.*, 2014 [121] | Thiazine Red aggregation, TEM, CD | Recombinant |
| Reynolds *et al.*, 2005 [122] | Right-angle laser light scattering, TEM | Recombinant |
| Yuzwa *et al.*, 2014 [119] | ThS, heparin-induced aggregation, filter-trap assay, WB | Recombinant |
| Mead *et al.,* 2016 [115] | ThS, fractionation experiments, confocal microscopy | Recombinant |
| Necula and Kuret, 2004 [116] | TEM, right angle laser light scattering | Recombinant |
| Chang *et al.,* 2011 [117] | Thiazine red aggregation, TEM, dot-blot | Recombinant |
| Yoshitake *et al.*, 2016 [120] | ThT, heparin-induced sedimentation assay, AFM | Recombinant |
| Luo *et al.*, 2014 [118] | WB, SDS-PAGE | Recombinant |
| Alpha-Synuclein | | |
| Bartels *et al.*, 2014 [123] | ThT, CD | Recombinant |
| Kang *et al.*, 2012 [124] | ThT, EM, CD | Recombinant |
| Bu *et al.*, 2017 [125] | Solid-state nanopores, REMD simulation | Semisynthetic |
| Birol *et al.*, 2019 [127] | Congo Red, TEM and PAGE | Recombinant |
| Oliveira *et al*., 2017 [126] | ThT, IHC | Recombinant |
| Sanyal *et al.*, 2019 [128] | ThT, WB, TEM | Recombinant |
| Vicente *et al.*, 2017 [129] | ThT, TEM, CD, SEC, dynamic light scattering | Recombinant |
| Qin *et al.*, 2006 [133] | ThT, CD, AFM, size exclusion HPLC, small angle x-ray scattering | Recombinant |
| Xiang *et al.*, 2013 [131] | ThT, WB, SEC, AFM, dot-blot | Recombinant |
| Xiang *et al.*, 2015 [132] | SDS-PAGE, WB, SEC, SDGC | Recombinant |
| Burai *et al.,* 2015 [139] | ThT, SEC, TEM, SDS-PAGE | Recombinant |
| Liu *et al.*, 2011 [140] | WB, SDS-PAGE, ThT, CD, EM | Recombinant |
| Hodara *et al.*, 2004 [141] | ThT, SDS-PAGE, EM, immunoelectron microscopy, CD | Recombinant |
| Souza e*t al.*, 2000 [142] | SDS-PAGE, WB, SEC | Recombinant |
| Levine *et al.*, 2019 [134] | ThT, TEM, proteinase-K digestion and SDS-PAGE, dynamic light scattering, CD | Recombinant and synthetic |
| Zhang *et al.*, 2017 [135] | ThT, WB, AFM | Synthetic |
| Marotta *et al.*, 2015 [136] | ThT, CD, dynamic light scattering, TEM, WB | Recombinant and synthetic |
| Fujiwara e*t al.*, 2002 [137] | ThT, TEM, SDS-PAGE | Recombinant, purified from human brain tissue |
| Samuel *et al.*, 2015 [138] | CD, Proteinase-K, WB, SDS-PAGE, EM | Recombinant |
| Krumova e*t al.*, 2011 [130] | ThT, TEM | Recombinant |
| TDP-43 | | |
| Wang *et al*., 2017 [143] | IHC, WB | Recombinant |
| Cohen *et al.*, 2015 [144] | IHC, WB | Recombinant |
| Zhang *et al.*, 2009 [145] | WB, IHC | Recombinant |
| Li *et al.*, 2011 [149] | IHC, SDS-PAGE, WB, filter trap assay | Recombinant |
| Carlomagno et al., 2014 [146] | ThS, EM | Recombinant |
| Hasegawa e*t al.*, 2008 [147] | Immunoelectron microscopy, WB, EM | Recombinant |
| Brady *et al.*, 2011 [148] | IHC, WB | Recombinant |
| SOD1 | | |
| Rasouli *et al.*, 2017 [152] | ThT, ANS aggregation, TEM, SDS-PAGE | Recombinant |
| Niikura e*t al.*, 2014 [153] | IHC, WB | Recombinant |
| Fei *et al.*, 2006 [154] | IHC, SDS-PAGE, WB | Recombinant |
| Huntingtin | | |
| Chaibva *et al.,* 2016 [155] | ThT, AFM | Synthetic |
| Chiki *et al.*, 2017 [156] | ThT, TEM, sedimentation assay, AFM, CD | Semisynthetic |
| Ansaloni *et al.*, 2014 [157] | ThT, AFM, TEM, CD | Semisynthetic |
| Cariulo *et al.*, 2017 [158] | Filter trap assay, TR-FRET immunoassay | Semisynthetic |
| DeGuire *et al.*, 2018 [159] | CD, AFM, TEM, ultra-HPLC | Semisynthetic |
| Gu *et al*.*,* 2009 [160] | HPLC Sedimentation Assay, EM, IHC | Synthetic |
| Lunkes e*t al.*, 2002 [161] | IHC | Recombinant |
| Ataxins | | |
| Ryu *et al.*, 2010 [163] | IHC, filter retardation assay | Recombinant |
| Haacke *et al.*, 2006 [164] | Filter retardation assay, SDS-PAGE, WB | Recombinant |
| Prion protein | | |
| Dear *et al.*, 2007 [165] | ThT, EM, Congo Red, CD, FT-IR spectroscopy | Recombinant |
| Giannopoulos *et al.*, 2009 [166] | TEM, WB, Congo Red, immunoelectron microscopy | Recombinant |

Abbreviations: AFM, atomic force microscopy; ANS, 1-anilinonaphthalene-8-sulfonate; CD, circular dichroism; EM, electron microscopy; FC, flow cytometry; FT-IR, Fourier-transform infra-red; HPLC, high performance liquid chromatography; IHC, immunohistochemistry; REMD, replica-exchange molecular dynamics; SDGC, sucrose density gradient centrifugation; SEC, size exclusion chromatography; SDS-PAGE, sodium dodecyl sulfate-polyacrylamide gel electrophoresis; TEM, transmission electron microscopy; ThS, thioflavin S assay; ThT, thioflavin T assay; TR-FRET, time-resolved fluorescence energy transfer; WB, western blotting.
